# Supplementary material for: Resolving the full spectrum of human genome variation using Linked-Reads
Source: Genome Res. 2019 Apr;29(4):635–45. doi: 10.1101/gr.234443.118 (PMC6442396; doi:10.1101/gr.234443.118)
Supplement: Supplemental Material [file supp_gr.234443.118_Supplemental_longranger-2.2.zip › longranger-2.2.tar.bz2!/lr2.2-public/lib/stan/cmdstan/stan/doc/api/html/classstan_1_1agrad_1_1var.html]

Stan: stan::agrad::var Class Reference


|  |  |
| --- | --- |
|  | Stan  2.5.0  probability, sampling & optimization |


- Main Page
- Related Pages
- Namespaces
- Classes
- Files

- Class List
- Class Index
- Class Hierarchy
- Class Members

All Classes Namespaces Files Functions Variables Typedefs Enumerations Enumerator Friends Macros Pages

- stan
- agrad
- var

Public Types |
Public Member Functions |
Public Attributes |
Friends |
List of all members

stan::agrad::var Class Reference

Independent (input) and dependent (output) variables for gradients.
More...

`#include <var.hpp>`

|  |  |
| --- | --- |
| Public Types | |
| typedef double | Scalar |
|  | |

|  |  |
| --- | --- |
| Public Member Functions | |
| bool | is\_uninitialized () |
|  | Return `true` if this variable has been declared, but not been defined. More... |
|  | |
|  | var (vari \*vi) |
|  | Construct a variable from a pointer to a variable implementation. More... |
|  | |
|  | var () |
|  | Construct a variable for later assignment. More... |
|  | |
|  | var (bool b) |
|  | Construct a variable by static casting the specified value to `double`. More... |
|  | |
|  | var (char c) |
|  | Construct a variable by static casting the specified value to `double`. More... |
|  | |
|  | var (short n) |
|  | Construct a variable by static casting the specified value to `double`. More... |
|  | |
|  | var (unsigned short n) |
|  | Construct a variable by static casting the specified value to `double`. More... |
|  | |
|  | var (int n) |
|  | Construct a variable by static casting the specified value to `double`. More... |
|  | |
|  | var (unsigned int n) |
|  | Construct a variable by static casting the specified value to `double`. More... |
|  | |
|  | var (long int n) |
|  | Construct a variable by static casting the specified value to `double`. More... |
|  | |
|  | var (unsigned long int n) |
|  | Construct a variable by static casting the specified value to `double`. More... |
|  | |
|  | var (unsigned long long n) |
|  | Construct a variable by static casting the specified value to `double`. More... |
|  | |
|  | var (long long n) |
|  | Construct a variable by static casting the specified value to `double`. More... |
|  | |
|  | var (float x) |
|  | Construct a variable by static casting the specified value to `double`. More... |
|  | |
|  | var (double x) |
|  | Construct a variable with the specified value. More... |
|  | |
|  | var (long double x) |
|  | Construct a variable by static casting the specified value to `double`. More... |
|  | |
| double | val () const |
|  | Return the value of this variable. More... |
|  | |
| double | adj () const |
|  | Return the derivative of the root expression with respect to this expression. More... |
|  | |
| void | grad (std::vector< var > &x, std::vector< double > &g) |
|  | Compute the gradient of this (dependent) variable with respect to the specified vector of (independent) variables, assigning the specified vector to the gradient. More... |
|  | |
| vari & | operator\* () |
|  | Return a reference to underlying implementation of this variable. More... |
|  | |
| vari \* | operator-> () |
|  | Return a pointer to the underlying implementation of this variable. More... |
|  | |
| var & | operator+= (const var &b) |
|  | The compound add/assignment operator for variables (C++). More... |
|  | |
| var & | operator+= (const double b) |
|  | The compound add/assignment operator for scalars (C++). More... |
|  | |
| var & | operator-= (const var &b) |
|  | The compound subtract/assignment operator for variables (C++). More... |
|  | |
| var & | operator-= (const double b) |
|  | The compound subtract/assignment operator for scalars (C++). More... |
|  | |
| var & | operator\*= (const var &b) |
|  | The compound multiply/assignment operator for variables (C++). More... |
|  | |
| var & | operator\*= (const double b) |
|  | The compound multiply/assignment operator for scalars (C++). More... |
|  | |
| var & | operator/= (const var &b) |
|  | The compound divide/assignment operator for variables (C++). More... |
|  | |
| var & | operator/= (const double b) |
|  | The compound divide/assignment operator for scalars (C++). More... |
|  | |

|  |  |
| --- | --- |
| Public Attributes | |
| vari \* | vi\_ |
|  | Pointer to the implementation of this variable. More... |
|  | |

|  |  |
| --- | --- |
| Friends | |
| std::ostream & | operator<< (std::ostream &os, const var &v) |
|  | Write the value of this auto-dif variable and its adjoint to the specified output stream. More... |
|  | |

## Detailed Description

Independent (input) and dependent (output) variables for gradients.

This class acts as a smart pointer, with resources managed by an agenda-based memory manager scoped to a single gradient calculation.

An agrad::var is constructed with a double and used like any other scalar. Arithmetical functions like negation, addition, and subtraction, as well as a range of mathematical functions like exponentiation and powers are overridden to operate on agrad::var values objects.

Definition at line 27 of file var.hpp.

## Member Typedef Documentation

typedef double stan::agrad::var::Scalar

Definition at line 31 of file var.hpp.

## Constructor & Destructor Documentation

|  |  |  |  |  |  |
| --- | --- | --- | --- | --- | --- |
| stan::agrad::var::var | ( | vari \* | *vi* | ) |  |

inlineexplicit

Construct a variable from a pointer to a variable implementation.

Parameters
:   |  |  |
    | --- | --- |
    | vi | Variable implementation. |

Definition at line 60 of file var.hpp.

|  |  |  |  |  |
| --- | --- | --- | --- | --- |
| stan::agrad::var::var | ( |  | ) |  |

inline

Construct a variable for later assignment.

This is implemented as a no-op, leaving the underlying implementation dangling. Before an assignment, the behavior is thus undefined just as for a basic double.

Definition at line 71 of file var.hpp.

|  |  |  |  |  |  |
| --- | --- | --- | --- | --- | --- |
| stan::agrad::var::var | ( | bool | *b* | ) |  |

inline

Construct a variable by static casting the specified value to `double`.

Parameters
:   |  |  |
    | --- | --- |
    | b | Value. |

Definition at line 81 of file var.hpp.

|  |  |  |  |  |  |
| --- | --- | --- | --- | --- | --- |
| stan::agrad::var::var | ( | char | *c* | ) |  |

inline

Construct a variable by static casting the specified value to `double`.

Parameters
:   |  |  |
    | --- | --- |
    | c | Value. |

Definition at line 91 of file var.hpp.

|  |  |  |  |  |  |
| --- | --- | --- | --- | --- | --- |
| stan::agrad::var::var | ( | short | *n* | ) |  |

inline

Construct a variable by static casting the specified value to `double`.

Parameters
:   |  |  |
    | --- | --- |
    | n | Value. |

Definition at line 101 of file var.hpp.

|  |  |  |  |  |  |
| --- | --- | --- | --- | --- | --- |
| stan::agrad::var::var | ( | unsigned short | *n* | ) |  |

inline

Construct a variable by static casting the specified value to `double`.

Parameters
:   |  |  |
    | --- | --- |
    | n | Value. |

Definition at line 111 of file var.hpp.

|  |  |  |  |  |  |
| --- | --- | --- | --- | --- | --- |
| stan::agrad::var::var | ( | int | *n* | ) |  |

inline

Construct a variable by static casting the specified value to `double`.

Parameters
:   |  |  |
    | --- | --- |
    | n | Value. |

Definition at line 121 of file var.hpp.

|  |  |  |  |  |  |
| --- | --- | --- | --- | --- | --- |
| stan::agrad::var::var | ( | unsigned int | *n* | ) |  |

inline

Construct a variable by static casting the specified value to `double`.

Parameters
:   |  |  |
    | --- | --- |
    | n | Value. |

Definition at line 131 of file var.hpp.

|  |  |  |  |  |  |
| --- | --- | --- | --- | --- | --- |
| stan::agrad::var::var | ( | long int | *n* | ) |  |

inline

Construct a variable by static casting the specified value to `double`.

Parameters
:   |  |  |
    | --- | --- |
    | n | Value. |

Definition at line 141 of file var.hpp.

|  |  |  |  |  |  |
| --- | --- | --- | --- | --- | --- |
| stan::agrad::var::var | ( | unsigned long int | *n* | ) |  |

inline

Construct a variable by static casting the specified value to `double`.

Parameters
:   |  |  |
    | --- | --- |
    | n | Value. |

Definition at line 151 of file var.hpp.

|  |  |  |  |  |  |
| --- | --- | --- | --- | --- | --- |
| stan::agrad::var::var | ( | unsigned long long | *n* | ) |  |

inline

Construct a variable by static casting the specified value to `double`.

Parameters
:   |  |  |
    | --- | --- |
    | n | Value. |

Definition at line 161 of file var.hpp.

|  |  |  |  |  |  |
| --- | --- | --- | --- | --- | --- |
| stan::agrad::var::var | ( | long long | *n* | ) |  |

inline

Construct a variable by static casting the specified value to `double`.

Parameters
:   |  |  |
    | --- | --- |
    | n | Value. |

Definition at line 171 of file var.hpp.

|  |  |  |  |  |  |
| --- | --- | --- | --- | --- | --- |
| stan::agrad::var::var | ( | float | *x* | ) |  |

inline

Construct a variable by static casting the specified value to `double`.

Parameters
:   |  |  |
    | --- | --- |
    | x | Value. |

Definition at line 181 of file var.hpp.

|  |  |  |  |  |  |
| --- | --- | --- | --- | --- | --- |
| stan::agrad::var::var | ( | double | *x* | ) |  |

inline

Construct a variable with the specified value.

Parameters
:   |  |  |
    | --- | --- |
    | x | Value of the variable. |

Definition at line 190 of file var.hpp.

|  |  |  |  |  |  |
| --- | --- | --- | --- | --- | --- |
| stan::agrad::var::var | ( | long double | *x* | ) |  |

inline

Construct a variable by static casting the specified value to `double`.

Parameters
:   |  |  |
    | --- | --- |
    | x | Value. |

Definition at line 200 of file var.hpp.

## Member Function Documentation

|  |  |  |  |  |
| --- | --- | --- | --- | --- |
| double stan::agrad::var::adj | ( |  | ) | const |

inline

Return the derivative of the root expression with respect to this expression.

This method only works after one of the `grad()` methods has been called.

Returns
:   Adjoint value for this variable.

Definition at line 221 of file var.hpp.

|  |  |  |  |
| --- | --- | --- | --- |
| void stan::agrad::var::grad | ( | std::vector< var > & | *x*, |
|  |  | std::vector< double > & | *g* |
|  | ) |  |  |

inline

Compute the gradient of this (dependent) variable with respect to the specified vector of (independent) variables, assigning the specified vector to the gradient.

The grad() function does *not* recover memory. In Stan 2.4 and earlier, this function did recover memory.

Parameters
:   |  |  |
    | --- | --- |
    | x | Vector of independent variables. |
    | g | Gradient vector of partial derivatives of this variable with respect to x. |

Definition at line 237 of file var.hpp.

|  |  |  |  |  |
| --- | --- | --- | --- | --- |
| bool stan::agrad::var::is\_uninitialized | ( |  | ) |  |

inline

Return `true` if this variable has been declared, but not been defined.

Any attempt to use an undefined variable's value or adjoint will result in a segmentation fault.

Returns
:   `true` if this variable does not yet have a defined variable.

Definition at line 51 of file var.hpp.

|  |  |  |  |  |
| --- | --- | --- | --- | --- |
| vari& stan::agrad::var::operator\* | ( |  | ) |  |

inline

Return a reference to underlying implementation of this variable.

If `x` is of type `var`, then applying this operator, `*x`, has the same behavior as `*(x.vi_)`.

*Warning*: The returned reference does not track changes to this variable.

Returns
:   variable

Definition at line 259 of file var.hpp.

|  |  |  |  |  |  |
| --- | --- | --- | --- | --- | --- |
| var & stan::agrad::var::operator\*= | ( | const var & | *b* | ) |  |

inline

The compound multiply/assignment operator for variables (C++).

If this variable is a and the argument is the variable b, then (a \*= b) behaves exactly the same way as (a = a \* b). Note that the result is an assignable lvalue.

Parameters
:   |  |  |
    | --- | --- |
    | b | The variable to multiply this variable by. |

Returns
:   The result of multiplying this variable by the specified variable.

Definition at line 10 of file operator\_multiply\_equal.hpp.

|  |  |  |  |  |  |
| --- | --- | --- | --- | --- | --- |
| var & stan::agrad::var::operator\*= | ( | const double | *b* | ) |  |

inline

The compound multiply/assignment operator for scalars (C++).

If this variable is a and the argument is the scalar b, then (a \*= b) behaves exactly the same way as (a = a \* b). Note that the result is an assignable lvalue.

Parameters
:   |  |  |
    | --- | --- |
    | b | The scalar to multiply this variable by. |

Returns
:   The result of multplying this variable by the specified variable.

Definition at line 15 of file operator\_multiply\_equal.hpp.

|  |  |  |  |  |  |
| --- | --- | --- | --- | --- | --- |
| var & stan::agrad::var::operator+= | ( | const var & | *b* | ) |  |

inline

The compound add/assignment operator for variables (C++).

If this variable is a and the argument is the variable b, then (a += b) behaves exactly the same way as (a = a + b), creating an intermediate variable representing (a + b).

Parameters
:   |  |  |
    | --- | --- |
    | b | The variable to add to this variable. |

Returns
:   The result of adding the specified variable to this variable.

Definition at line 10 of file operator\_plus\_equal.hpp.

|  |  |  |  |  |  |
| --- | --- | --- | --- | --- | --- |
| var & stan::agrad::var::operator+= | ( | const double | *b* | ) |  |

inline

The compound add/assignment operator for scalars (C++).

If this variable is a and the argument is the scalar b, then (a += b) behaves exactly the same way as (a = a + b). Note that the result is an assignable lvalue.

Parameters
:   |  |  |
    | --- | --- |
    | b | The scalar to add to this variable. |

Returns
:   The result of adding the specified variable to this variable.

Definition at line 15 of file operator\_plus\_equal.hpp.

|  |  |  |  |  |  |
| --- | --- | --- | --- | --- | --- |
| var & stan::agrad::var::operator-= | ( | const var & | *b* | ) |  |

inline

The compound subtract/assignment operator for variables (C++).

If this variable is a and the argument is the variable b, then (a -= b) behaves exactly the same way as (a = a - b). Note that the result is an assignable lvalue.

Parameters
:   |  |  |
    | --- | --- |
    | b | The variable to subtract from this variable. |

Returns
:   The result of subtracting the specified variable from this variable.

Definition at line 10 of file operator\_minus\_equal.hpp.

|  |  |  |  |  |  |
| --- | --- | --- | --- | --- | --- |
| var & stan::agrad::var::operator-= | ( | const double | *b* | ) |  |

inline

The compound subtract/assignment operator for scalars (C++).

If this variable is a and the argument is the scalar b, then (a -= b) behaves exactly the same way as (a = a - b). Note that the result is an assignable lvalue.

Parameters
:   |  |  |
    | --- | --- |
    | b | The scalar to subtract from this variable. |

Returns
:   The result of subtracting the specified variable from this variable.

Definition at line 15 of file operator\_minus\_equal.hpp.

|  |  |  |  |  |
| --- | --- | --- | --- | --- |
| vari\* stan::agrad::var::operator-> | ( |  | ) |  |

inline

Return a pointer to the underlying implementation of this variable.

If `x` is of type `var`, then applying this operator, `x->`, behaves the same way as `x.vi_->`.

*Warning*: The returned result does not track changes to this variable.

Definition at line 273 of file var.hpp.

|  |  |  |  |  |  |
| --- | --- | --- | --- | --- | --- |
| var & stan::agrad::var::operator/= | ( | const var & | *b* | ) |  |

inline

The compound divide/assignment operator for variables (C++).

If this variable is a and the argument is the variable b, then (a /= b) behaves exactly the same way as (a = a / b). Note that the result is an assignable lvalue.

Parameters
:   |  |  |
    | --- | --- |
    | b | The variable to divide this variable by. |

Returns
:   The result of dividing this variable by the specified variable.

Definition at line 10 of file operator\_divide\_equal.hpp.

|  |  |  |  |  |  |
| --- | --- | --- | --- | --- | --- |
| var & stan::agrad::var::operator/= | ( | const double | *b* | ) |  |

inline

The compound divide/assignment operator for scalars (C++).

If this variable is a and the argument is the scalar b, then (a /= b) behaves exactly the same way as (a = a / b). Note that the result is an assignable lvalue.

Parameters
:   |  |  |
    | --- | --- |
    | b | The scalar to divide this variable by. |

Returns
:   The result of dividing this variable by the specified variable.

Definition at line 15 of file operator\_divide\_equal.hpp.

|  |  |  |  |  |
| --- | --- | --- | --- | --- |
| double stan::agrad::var::val | ( |  | ) | const |

inline

Return the value of this variable.

Returns
:   The value of this variable.

Definition at line 209 of file var.hpp.

## Friends And Related Function Documentation

|  |  |  |  |
| --- | --- | --- | --- |
| std::ostream& operator<< | ( | std::ostream & | *os*, |
|  |  | const var & | *v* |
|  | ) |  |  |

friend

Write the value of this auto-dif variable and its adjoint to the specified output stream.

Parameters
:   |  |  |
    | --- | --- |
    | os | Output stream to which to write. |
    | v | Variable to write. |

Returns
:   Reference to the specified output stream.

Definition at line 388 of file var.hpp.

## Member Data Documentation

vari\* stan::agrad::var::vi\_

Pointer to the implementation of this variable.

This value should not be modified, but may be accessed in `var` operators to construct `vari` instances.

Definition at line 40 of file var.hpp.

---

The documentation for this class was generated from the following files:

- src/stan/agrad/rev/var.hpp
- src/stan/agrad/rev/operators/operator\_divide\_equal.hpp
- src/stan/agrad/rev/operators/operator\_minus\_equal.hpp
- src/stan/agrad/rev/operators/operator\_multiply\_equal.hpp
- src/stan/agrad/rev/operators/operator\_plus\_equal.hpp


---

[ Stan Home Page ]

*© 2011–2014,
Stan Development Team.*
